# Unanticipated questions can yield unanticipated outcomes in investigative interviews

**DOI:** 10.1371/journal.pone.0208751

**Published:** 2018-12-07

**Authors:** Tom Parkhouse, Thomas C. Ormerod

**Affiliations:** Department of Psychology, University of Sussex, Brighton, East Sussex, United Kingdom; University of Padova, ITALY

## Abstract

Asking unanticipated questions in investigative interviews can elicit differences in the verbal behaviour of truth-tellers and liars: When faced with unanticipated questions, liars give less detailed and consistent responses than truth-tellers. Do such differences in verbal behaviour lead to an improvement in the accuracy of interviewers’ veracity judgements? Two empirical studies evaluated the efficacy of the unanticipated questions technique. Experiment 1 compared two types of unanticipated questions (questions regarding the planning of a task and questions regarding the specific spatial and temporal details associated with the task), assessing the veracity judgements of interviewers and verbal content of interviewees’ responses. Experiment 2 assessed veracity judgements of independent observers. Overall, the results provide little support for the technique. For interviewers, unanticipated questions failed to improve veracity judgement accuracy above chance. Reality monitoring analysis revealed qualitatively distinct information in the responses to the two unanticipated question types, though little distinction between the responses of truth-tellers and liars. Accuracy for observers was greater when judging transcripts of unanticipated questions, and this effect was stronger for spatial and temporal questions than planning questions. The benefits of unanticipated questioning appear limited to post-interview situations. Furthermore, the type of unanticipated question affects both the type of information gathered and the ability to detect deceit.

## Introduction

Bond et al’s [1] influential meta-analysis of deception detection reached a worrying conclusion: individuals, regardless of training or experience, are generally poor at distinguishing between truth and lies. Analysing the accuracy of veracity judgements made across 206 studies involving over 20,000 judges, the authors found an overall accuracy rate of just 54%, in part because the general public and trained experts alike appear erroneously to put their faith in non-verbal indicators of deception [2,3]. DePaulo et al’s [4] meta-analysis revealed that statements made by liars were less consistent, less coherent, and contained fewer details than those given by truth-tellers. Thus, recent research has focussed on verbal behaviours such as differences in response length, level of detail and consistency, as cues to deceit [5–7]. The unanticipated questions technique [7], evaluated in this paper, is designed to emphasise differences in verbal behaviours of truth-tellers and liars.

Asking questions that an interviewee has not anticipated should, according to Vrij [8], increase a liar’s cognitive load, resulting in observable differences in their verbal behaviours compared to those of an honest interviewee. Research has shown that liars give less detailed, less plausible, and/or less consistent answers in response to unanticipated questions than truth-tellers [9,10]. Some interesting new work has shown that unanticipated questions may even be successfully utilised to detect false information being provided electronically, showing that liars exhibit certain cues to deceit, such as prolonged mouse trajectory, when answering unanticipated questions [11, 12]. Numerous studies have investigated the efficacy of the unanticipated questions approach in distinguishing between truth-tellers and liars [13,14]. A recent meta-analysis showed that the cognitive approach to lie detection, which makes use of unanticipated questions, led to an overall detection accuracy rate of 71% [15]. Furthermore, unanticipated questioning is one of the six principles of the Controlled Cognitive Engagement interview technique, arguably one of the most successful practical methods for detecting deception developed for field use [16] and has been recommended as best practice in intelligence interviewing [17].

The majority of studies investigating the efficacy of the approach have focussed on post-hoc analyses of interviewees’ verbal behaviour (e.g., statement consistency, level of detail, etc.), as opposed to real-time veracity judgements made by interviewers. Research to date has not addressed whether effects of asking unanticipated questions are noticeable to the interviewer. The present study was designed to evaluate the unanticipated questions technique, specifically whether its efficacy extends to real-time veracity judgements.

The unanticipated questions approach, it is suggested, exploits differences in the cognitive load faced by truth-tellers and liars. It is well established that telling a lie imposes greater cognitive load on the individual than telling the truth [8,18]. Results from fMRI studies have shown that lying, compared to truth telling, is associated increased neural activity in the prefrontal cortex, an area often linked to cognitive engagement [19,20]. There are a number of reasons why lying is more cognitively demanding than telling the truth. For example, in an interview, a liar must present their false account while simultaneously inhibiting the truth [21]. Additionally, liars are more likely to monitor and control their own outward behaviour, while also attempting to decipher the behaviour of the interviewer, which again increases their cognitive load [22]. Interview techniques that increase the cognitive load faced by liars have been shown to improve veracity judgement accuracy rates [23].

Given the opportunity, liars plan how they will behave and what they will say when interviewed [24]. As part of this planning, they are likely to anticipate questions they may be asked and prepare suitable responses to them, developing a ‘lie script’ [25,26]. However, planning will only help if they correctly anticipate the questions that are asked. By asking unanticipated questions, the interviewer breaks the lie script and forces liars to answer spontaneously, which should increase their cognitive load and change their verbal behaviour [8]. In contrast, an interviewee telling the truth should have less difficulty providing a response to unanticipated questions because they can rely on real memories of events. Accordingly, Vrij [8] states that ‘truth-tellers experience similar levels of cognitive load while answering expected and unexpected questions, and they should produce more comparable answers to the expected and unexpected questions than liars.’ (p. 187).

Clemens et al [27] argue that when liars are formulating their lie script, they tend to prepare for questions that focus on their intentions (e.g., “What items did you intend to purchase whilst at the shopping centre today?”) and fail to prepare for questions about the planning of these intentions (e.g., “Tell me about the order in which you planned to purchase these items”). Sooniste et al [28] had participants plan either a non-criminal (truth-tellers) or a mock-criminal (liars) act. Liars were further instructed to prepare a cover story to mask their criminal intentions. In subsequent interviews, both groups of participants were asked questions concerning their intentions and the planning of their intentions. The planning questions were rated as significantly less anticipated than the intentions questions. Furthermore, truth-tellers’ responses to the planning questions were shown to include significantly more detail than liars’ responses, with no such difference occurring in response to questions on their intentions. This supports the idea that unanticipated questions give rise to noticeable differences in the verbal behaviour of truth-tellers and liars, and subsequent studies have reported similar differences [9,14].

Other studies have focussed on spatial and temporal details as the basis for unanticipated questions [29,30]. Vrij et al [7] asked pairs of participants to either tell the truth or lie about having lunch together. They asked general questions about the task that might be anticipated (e.g. “Can you tell me in as much detail as possible what you did while you were in the restaurant?”), as well as specific spatial and temporal detail questions (e.g. “In relation to the front door, where did you and your friend sit?”; “How long was it between the staff taking your order and you receiving your food?”). Participants rated spatial and temporal questions as less anticipated than the general questions. Moreover, statements provided by lying pairs were less consistent than honest pairs, but only when answering the spatial and temporal questions. Liars’ responses contained less detail than truth tellers’ responses across all question types, and this difference was more pronounced in the spatial and temporal questions. This type of questioning has subsequently been applied to individual interviewees with similar findings [13].

Although the unanticipated questions approach has received considerable support in terms of its ability to distinguish true and false accounts on the basis of verbal cues [7,13], there have been some inconsistent findings. Vrij et al [31] found that, while liars gave less detailed answers to anticipated questions, there was no difference between truth-tellers and liars in the amount of detail provided in response to unanticipated questions. One potential reason for these mixed findings is variability in the types of unanticipated question used across studies. Knieps et al [32] asked interviewees unexpected questions about the occurrence of a mental image they may have had during their planning of a mock criminal event; Vrij et al [10] required interviewees to provide a sketch of their workplace; while Warmelink et al [14] interviews included unanticipated questions about transportation. Furthermore, Warmelink et al [33] introduced the idea of familiar and unfamiliar lies, with unanticipated questions regarding the background and details associated with interviewees’ occupations. In general, studies have focussed either on questions regarding the planning of an event or on spatial and temporal details associated with an event. Although it is reasonable to imagine that different forms of unanticipated question will elicit qualitatively distinct responses, no study has compared them directly.

The majority of unanticipated question studies comprise post-hoc analyses of interviewees’ verbal behaviour, looking at differences in the level of detail, consistency and statement length [7,9,14,34]. Vrij et al [30,31] conducted follow-up studies where observers made veracity judgements from interview transcripts, finding that accuracy was greater than chance only with transcripts containing unanticipated questions. However, no studies have required interviewers to make real-time veracity judgements. The goal of many investigative interviews (e.g., interrogations in the US justice system, security screening, and vetting interviews) is to allow the interviewer to establish the veracity of the interviewee’s account. In a study by Sooniste et al [35], experienced police officers were trained to detect deception using, among other methods, unanticipated questions. Subsequently, they interviewed truth-tellers and liars and were required to make real-time veracity judgements. The officers who were trained performed better than untrained officers, though this difference in accuracy was not significant. However, they were given the freedom to conduct the interview as they chose and so it was only possible to measure the presence of unanticipated questions in a post-hoc fashion.

Unanticipated questions may elicit verbal cues to deceit, but their effects on judgements of the interviewer are unknown. Vrij et al’s [15] meta-analysis into the cognitive approach to lie detection, which uses unanticipated questions, found across studies that veracity was correctly classified 71% of the time when using this technique, compared with only 56% using standard interview approaches. However, Levine et al [36] recently challenged these findings, arguing that the meta-analysis confounded dependent variables by combining human veracity detection rates and statistical classifications based on coded differences in interview transcripts. By re-examining the data, they showed a difference in accuracy rates obtained by the two outcome measures, with higher rates observed for statistical classifications (78%) than human judgements (62%). Therefore, it remains unclear whether statistical differences in verbal behaviour translate to an improvement in human veracity judgement accuracy.

The studies presented below examined the effects of unanticipated questions using three different empirical approaches. Experiment 1 provided a within-experiment comparison of the effectiveness of unanticipated planning and unanticipated spatial/temporal questions, to determine if the use of unanticipated questions leads to improved accuracy in the real-time veracity judgements made by interviewers. The resulting interviews were analysed using the Reality Monitoring (RM) framework [37] to examine whether anticipated and unanticipated questions generate differences in verbal content of truth-tellers’ and liars’ responses. In Experiment 2, transcripts of the interviews conducted in Experiment 1 were shown to a separate group of observers, who were required to make a veracity judgement.

## Experiment 1

In this experiment, truth-tellers completed a task which involved navigating around a university campus, while liars had to pretend to have conducted the same task. All interviewees were subsequently told to convince an interviewer that they had carried out the task. The interview questions were either questions that might be anticipated by interviewees (e.g., “What task did you carry out around the campus today?”), unanticipated questions about the planning of the task (e.g., “Please describe any changes you made to your plan during the planning stage”), or unanticipated questions regarding spatial and temporal details (e.g., “In building B, where were the boxes in relation to the door you entered through?”). Immediately following the interviews, interviewers made a veracity judgement concerning the interviewee’s account and were asked what information they based their decision on.

Based on previous work by Vrij and colleagues showing unanticipated questions in interviews results in differences in the verbal behaviour of truth-tellers and liars [13,14], interviewers should make more accurate veracity judgements when asking questions regarding planning or spatial and temporal details that are unlikely to be anticipated by interviewees than when asking the general questions about the event that are likely to be anticipated (Hypothesis 1). The unanticipated questions approach is grounded in the idea that liars will experience an increase in cognitive load when answering unanticipated questions compared to ones they have anticipated, while truth-tellers should experience similar levels across question type [8]. As such, liars should give higher ratings of cognitive complexity to the interviews involving unanticipated questions than the anticipated questions, with no such differences observed between the ratings given by truth-tellers (Hypothesis 2). Finally, given that the unanticipated questions approach is said to elicit differences in the verbal content of truth-tellers’ and liars’ accounts [8], interviewers who reported verbal content as the basis for their decisions should show greater judgement accuracy (Hypothesis 3). A failure to find support for each of these hypotheses would cast doubt upon the unanticipated questions framework.

The experiment also investigated differences in the verbal responses provided by truth tellers and liars, and whether they are amplified by asking unanticipated questions. The Reality Monitoring (RM) framework [37] asserts that an individual’s memory of a genuine experience is intrinsically associated with perceptual processes, meaning they will be richer in details related to sensory information (e.g., visual and auditory), contextual information (e.g., spatial and temporal) and affective information (e.g., references to emotional state) [38]. Accounts of imagined experiences are conceived endogenously, without any genuine perceptual information, meaning they are likely to be richer than accounts of genuine experiences in cognitive operations (e.g., references to thought processes) [39]. RM has been utilised in deception research, with several studies reporting it can distinguish between true and false accounts [38,40,41].

Unanticipated questions are designed to force the interviewee into providing a spontaneous, unprepared answer and as such a dishonest interviewee should have less opportunity to access related experience from memory [8]. Research has shown that unanticipated questions emphasise differences in truth-tellers’ and liars’ verbal behaviour in terms of statement length and level of detail [28]. These amplified differences should be detected by RM. Although there has been variation among studies that have utilised RM in terms of the linguistic categories used, the four most commonly associated with deception are words relating to sensory information (e.g. “saw”, “heard”), contextual information (e.g. “up”, “after”), affective information (e.g. “upset”, “pleased”), and cognitive mechanisms (e.g. “thought”, “considered”). Previous research has shown that truth tellers tend to use more sensory and contextual information words than liars [41] given that they have a true episodic memory of the event in question, which is likely to be rich in perceptual information [38]. Liars, on the other hand, have been shown to use more words related to cognitive mechanisms than truth tellers [40] because they must rely on imagined experience of the event, without genuine perceptual information [39]. Research on the affective information category is less clear. The original theory on which RM is based states that truth-tellers should use more affective information words than liars [37], and this pattern has previously been reported [42]. However, some findings show no difference between truth tellers and liars [40,41].

The number of words falling into the four RM categories was measured for each interview transcript using the linguistic analysis software LIWC [43]. Based on RM theory [37] and previous findings specific to deception [40–42], truth tellers should use more words associated with sensory, contextual and affective information and liars should use more words associated with cognitive mechanisms than truth tellers (Hypothesis 4). Additionally, based on the findings of Vrij and colleagues regarding the unanticipated questions approach [7,13], differences in the verbal content of truth tellers’ and liars’ responses should be amplified by the use of unanticipated questions (Hypothesis 5).

## Method

### Participants

#### Interviewees

Sixty interviewees were assigned to the truth-teller condition. Of these, 42 were female (M_*age*_ = 21.52, SD = 4.32) and 18 were male (M_*age*_ = 23.00, SD = 6.38). A further 60 interviewees were assigned to the liar condition. Of these, 47 were female (M_*age*_ = 20.38, SD = 2.65) and 13 were male (M_*age*_ = 22.69, SD = 4.23). Interviewees were UG and PG students recruited from a range of science and arts disciplines at the University of Sussex. Interviewees received either course credits or £5 for taking part. As an additional incentive, they were told that they would receive a further £5 if they were successful in convincing the interviewer that they were telling the truth. In reality, all interviewees received this extra money regardless of performance. This study was approved by the Sciences & Technology Cross-Schools Research Ethics Committee at the University of Sussex. All participants provided written consent.

#### Interviewers

Six female (M_*age*_ = 29.67, SD = 5.09) and four male (M_*age*_ = 30.75, SD = 10.91) Psychology doctoral students at the University of Sussex were selected to carry out the interviews. All attended training which comprised classroom-based instruction and practical exercises on using the interview protocol devised for this research, which consisted of a fixed set of questions varying by condition (S1 Appendix). Interviewers were given basic information about the task that the interviewees were going to be carrying out, but all were blind to the veracity of the interviewees and hypotheses of the study. Each conducted twelve interviews and was paid £65 for taking part.

### Design

A between-groups design was employed, with interviewees randomly assigned to either truth-teller (*n* = 60) or liar (*n* = 60) conditions. Interviewees were further randomly assigned to one of three interview conditions: anticipated (*n* = 40), planning (*n* = 40), or spatial/temporal (*n* = 40). Assignment was balanced across condition so that for each of the interview conditions, half were truth-tellers and half were liars.

### Procedure

#### Truth-tellers

Those assigned to the truth-teller condition arrived at the interview room and, after reading an information sheet and signing a consent form, were escorted to a room in another building on campus, where they received written instructions. The instructions informed the participant that they were currently in Room A and that in front of them they would see a stack of paper box files, each a different colour. In each trial, the number of boxes left in Room A was varied between two and four in order to prevent the interviewers being able to determine veracity based on the number of boxes left at Room A. The goal was to ensure that there were five boxes stacked in Room A by the end of the task, so interviewees should collect further boxes from Room B, located within another building on a remote side of the campus that is not frequented by anyone other than maintenance staff. They were also informed that the entrance to Room B had an access code and that, although one of the experimenters should be there to let them in, they should consider alternative routes in case the experimenter was unable to be there. In reality, the experimenter was always there to let them in. This instruction was included in order to create a scenario which would require a degree of forward planning by interviewees, and to introduce a degree of ambiguity to prevent interviewers from learning task-induced differences between truth-teller and liar accounts. They were instructed to take five minutes to plan how they would complete the task and then no more than 30 minutes to complete it and then return to the interview room. In order to encourage interviewees to spend time planning the task, the instructions again stated that they should consider both the time limit and the possibility that they would be unable to enter Room B via the main entrance. Prior to the interview following the task, they were instructed to answer all questions as accurately and honestly as possible. Interviewees were given a campus map that highlighted Room A and Room B. Interviewees kept track of time using their watch or phone.

#### Liars

Liars were informed that they would not be carrying out the navigation task but that their goal was to convince the interviewer that they had, and that they would have to answer interview questions dishonestly. They were given instructions for the navigation task, which were the same as the instructions given to truth-tellers, including the information regarding the potential complications accessing Room B, and the map of the campus. They were given five minutes planning time to develop a convincing story that would help them answer the interviewer’s questions.

#### The interview

Prior to each interview, the interviewer was handed one of three question lists and then was introduced to the interviewee. The experimenter turned on the two cameras (one directed at the interviewee and one at the interviewer) and then left the room, leaving the interviewer to ask the set of questions. Each of the question lists consisted of ten questions. The first five questions were the same in each list and consisted of general questions about the task that interviewees might have anticipated, such as “What task did you carry out around the campus today?” and “Describe the route you took from Room A to Room B.” The remaining questions differed according to condition: In the general condition, they were further general questions similar to the first five, such as “How many boxes were there in Room B?” In the planning condition, questions (adapted from those asked by Sooniste et al [2]) focussed on the planning of the task, such as “Explain what steps you would have taken had you not been able to access Room B via the main door” and “Please describe any changes you made to your plan during the planning stage.” In the spatial/temporal condition, they focussed on spatial and temporal details, such as “Try to imagine the layout and features of the Room B. Please describe this room, and be as detailed as you can” and “Please describe the task in full, but now in reverse order.”

In order to prevent the interviewers from gaining advantageous information as the experiment progressed, (e.g., that an experimenter was always in place at Room B), they were never given feedback on their performance until all twelve interviews had been completed.

#### Post-interview questionnaires

When the interview was complete, the interviewee completed two questionnaires. The first listed the ten questions that they had been asked and required them to state, using a 7-point Likert scale, how much they had anticipated each question prior to interview. The second gathered information, again using 7-point Likert scales, regarding how deceptive/truthful they had been, how cognitively demanding they found the interview, and how motivated they were to comply with the instructions.

The interviewers also completed a questionnaire after each interview in which they indicated whether they felt the interviewee had been lying or telling the truth, firstly on a 7-point Likert scale and secondly using a dichotomous forced choice decision. The questionnaire also required them to explain any verbal or non-verbal information they had based their decision on. Responses were subsequently coded as one of four categories: Verbal Content, such as “specific details in responses” or “consistency in responses”; Verbal Delivery, such as “tone of voice” or “responses seeming rehearsed”; Non-verbal Passive, such as “nervous demeanour” or “level of confidence”; and Non-verbal Active, such as “hand movements”, “body language” or “eye contact”.

## Results

### Manipulation checks

#### Interviewee compliance

Interviewees were asked to rate the extent to which they had been deceptive in the interview on a seven-point Likert scale (1 = totally truthful; 7 = totally deceptive). Interviewees assigned to liar conditions reported being more deceptive (M = 6.27, SD = 0.86) than those in the truth-teller condition (M = 1.15, SD = 0.71), *t* (118) = -35.54, *p* < .001, *d* = 6.49, 95% CI [5.56, 7.34]. Motivation to comply was high in both groups, with no difference in ratings between truth tellers (M = 6.08, SD = 1.05) and liars (M = 6.10, SD = 0.86), *t* (118) = -0.10, *p* = .93.

#### Interviewer compliance

Transcripts of the interviews were assessed to establish whether the interviewers had adhered to the interview protocol. The total number of deviations from the 10-question script was calculated for each interview. Deviations included missing out a question, changing the order of the questions, altering the wording of a question, asking an incomplete question, or asking an additional question. Overall, the number of deviations in each interview was low (M = 0.80, SD = 1.12) and the majority were due to interviewers slightly rephrasing questions to help the interviewee understand. A 2 (veracity: truth-teller or liar) × 3 (question type: anticipated, unanticipated planning, or unanticipated spatial/temporal) between-groups ANOVA showed no main effect of veracity, *F* (1, 114) = 2.13, *p* = .15, nor a main effect of question type, *F* (1, 114) = 0.41, *p* = .66. There was also not a significant veracity × question type interaction, *F* (1, 114) = 0.38, *p* = .69.

#### Anticipation

Interviewees rated the extent to which they had anticipated each question on a seven-point scale (1 = completely expected; 7 = completely unexpected). Mean anticipation was calculated for the final five questions of each interview. A 2 (veracity: truth-teller or liar) × 3 (question type: anticipated, unanticipated planning, unanticipated spatial/temporal) between-groups ANOVA showed no main effect of veracity, *F* (1, 114) = 0.95, *p* = .33, nor a significant veracity × question type interaction, *F* (2, 114) = 1.45, *p* = .24. There was a significant main effect of question type, *F* (2, 114) = 20.83, *p* < .001, $\eta_{p}^{2}=.27$, 95% CI [.13, .38]. Planned contrasts revealed that questions assigned to the anticipated conditions (M = 4.11, SD = 1.24) were significantly more anticipated than questions assigned to the unanticipated conditions (i.e., the average of planning and spatial/temporal questions combined; M = 5.36, SD = 0.87), *F* = 41.30, *p* < .001, *d* = 1.24, 95% CI [0.82, 1.64]. However, there was no significant difference in anticipation of planning questions (M = 5.43, SD = 0.85) and spatial/temporal questions (M = 5.30, SD = 0.89), *F* = 0.36, p = .55.

### Accuracy

#### Forced choice

The interviewer made a dichotomous decision post-interview regarding the veracity of each interviewee, and did so for two interviewees in each of the six conditions. The overall mean accuracy was 54%. A one-sample *t*-test showed that this was not significantly different from chance (50% correct), *t* (119) = 0.91, *p* = .36. In a series of one-sample t-tests (see Table 1) accuracy when asking anticipated questions was significantly better than chance at identifying truth-tellers, *t* (19) = 2.52, *p* = .021, *d* = 0.56, 95% CI [0.08, 1.03]. However, performance was significantly worse than chance at identifying liars, *t* (19) = -3.27, *p* = .004, *d* = 0.73, 95% CI [0.23, 1.22]. For truth-tellers and liars combined, performace was not significantly different from chance, *t* (39) = -0.31, *p* = .76.With unanticipated planning questions, performance did not differ from chance at interviewing truth-tellers, *t* (19) = 0.89, *p* = .39, liars, *t* (19) = -0.89, *p* = .39, or for truth-tellers and liars combined, *t* (39) = 0, *p* = 1. With unanticipated spatial/temporal questions, interviewers were significant better than chance at identifying truth-tellers, *t* (19) = 2.52, *p* = .021, *d* = 0.56, 95% CI [0.08, 1.03], but not at identifying liars, *t* (19) = 0.44, *p* = .67. For truth-tellers and liars combined, interviewer accuracy with the unanticipated spatial/temporal questions was also not significantly greater than chance, *t* (39) = 1.96, *p* = .06.

**Table 1:** Mean (SD) accuracy rates across each question type for both truth-tellers, liars, and overall.

Question Type	Truth-teller	Liar	Overall
Anticipated	**75%** (44%)	**20%** (41%)	48% (51%)
Planning	60% (50%)	40% (50%)	50% (51%)
Spatial/Temporal	**75%** (44%)	55% (51%)	65% (48%)

Note: Bold figures indicate that the accuracy significantly differed from chance (50%)

To investigate the relative effects of veracity and question type on the interviewers’ dichotomous judgement accuracy (where scores varied between 0 and 2, interviewers contributing two judgements in each condition), a 2 (veracity: truth-teller or liar) × 3 (question type: anticipated, unanticipated planning, or unanticipated spatial/temporal) repeated measures ANOVA was conducted. There was a significant effect of Veracity, *F* (1, 9) = 13.05, *p* = .006, $\eta_{p}^{2}=.59$, 95% CI [.08, .77], with overall accuracy greater for truth-tellers (70%) than for liars (38%). Neither Question Type, *F* (2, 8) = 1.56, *p* = .27, nor the interaction between Veracity and Question Type, *F* (2, 8) = 2.45, *p* = .15, was significant.

#### Veracity scale

**Fig 1:**
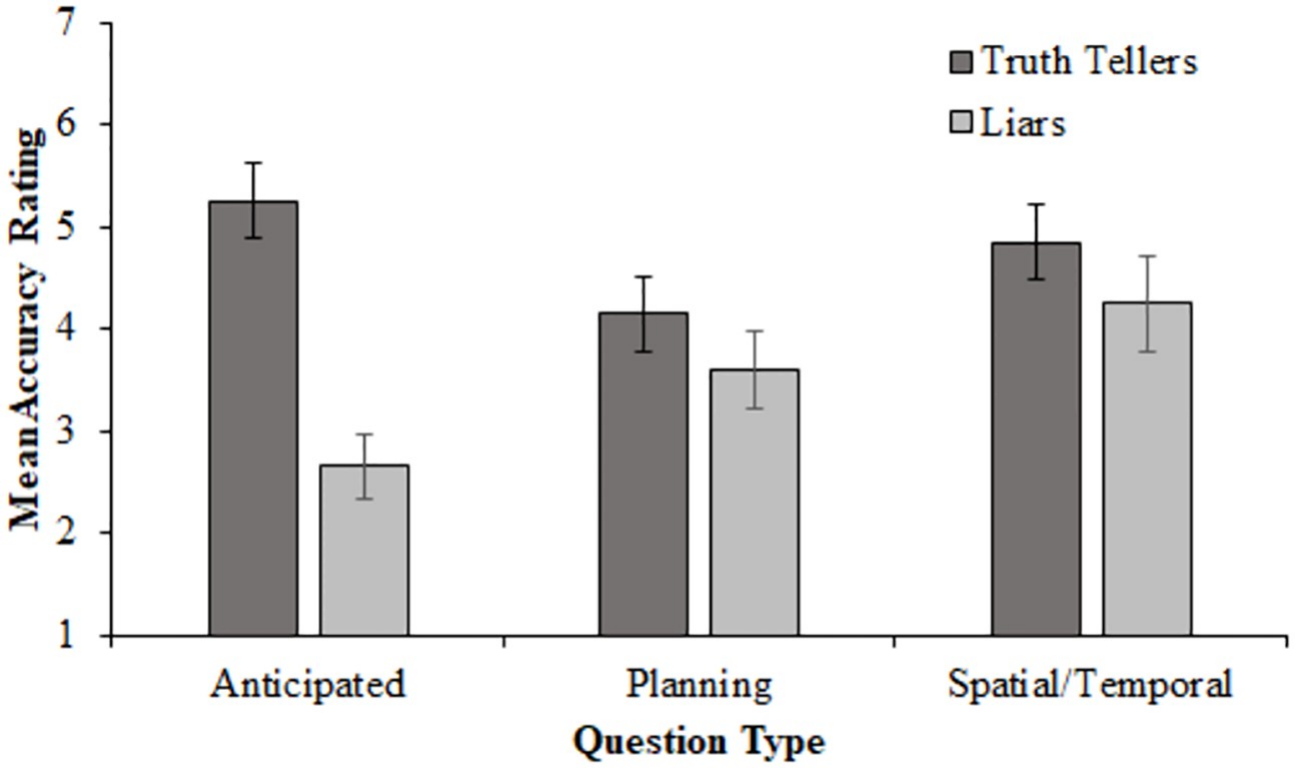
Mean interviewer accuracy (measured via 7-point Likert scale) across question type for truth-tellers and liars separately. Error bars represent +/- 1 SEM.

Interviewers were also asked to rate the extent to which they thought the interviewee was telling the truth or lying on a seven-point scale (1 = definitely lying; 7 = definitely telling the truth). Scores in the liar conditions were reversed so that higher scores indicate greater accuracy. Fig 1 shows the mean scores given across the three interview types for truth-tellers and liars.

To investigate the effects of veracity and question type, a 2 (veracity: truth-teller or liar) × 3 (question type: anticipated, unanticipated planning, or unanticipated spatial/temporal) between-groups ANOVA was performed on level of accuracy. There was a significant main effect of veracity, *F* (1, 114) = 16.10, *p* = < .001, $\eta_{p}^{2}=.12$, 95% CI [.03, .24], as well as a significant interaction, *F* (2, 114) = 4.70, *p* = .011, $\eta_{p}^{2}=.08$, 95% CI [.004, .17]. There was no main effect of question type, *F* (2, 114) = 1.88, *p* = .157. Planned contrasts reveal that accuracy was greater for truth-tellers (M = 4.75, SD = 1.67) than for liars (M = 3.50, SD = 1.86), *F* = 16.10, *p* < .001, *d* = 0.71, 95% CI [0.32, 0.93]. The difference in accuracy between truth-tellers and liars was significantly greater for the anticipated questions (M_*diff*_ = 2.60, SD = 3.08) than the two unanticipated question types combined (M_*diff*_ = 0.56, SD = 3.59), *F* = 9.36, *p* = .003, *d* = 0.61. However, there was no difference between the unanticipated spatial/temporal and unanticipated planning questions, *F* = 0.01, p = .95.

### Cognitive demand

**Fig 2:**
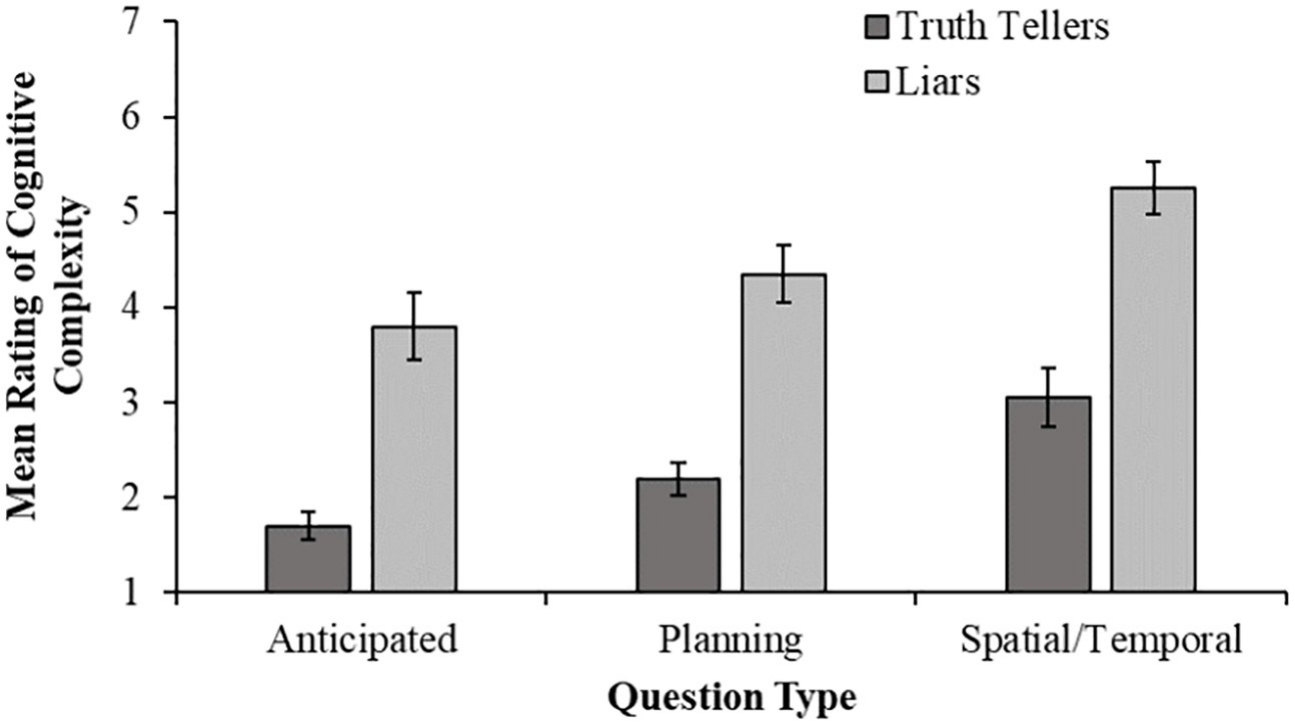
Mean ratings of cognitive complexity (1 = very easy, 7 = very difficult) given to each interview type for truth-tellers and liars separately. Error bars represent +/-1 SEM.

Interviewees were asked to rate how cognitively demanding they found the interview on a seven-point scale (1 = very easy; 7 = very difficult). Fig 2 shows the mean ratings given to each question type for truth-tellers and liars. To investigate the effects of veracity and question type, a 2 (veracity: truth-teller or liar) × 3 (question type: anticipated, unanticipated planning, unanticipated spatial/temporal) between-groups ANOVA was conducted on cognitive demand ratings. There were main effects of both veracity, *F* (1, 114) = 95.32, *p* < .001, $\eta_{p}^{2}=.46$, 95% CI [.32, .56] and question type, *F* (2, 114) = 13.75, *p* < .001, $\eta_{p}^{2}=.19$, 95% CI [.07, .31]. However, there was no significant interaction, *F* (2, 114) = 0.02, *p* = .98. Planned comparisons revealed that, overall, liars found the interviews more difficult (M = 4.47, SD = 1.49) than the truth-tellers (M = 2.32, SD = 1.13), *F* = 95.32, *p* < .001, *d* = 1.63, 95% CI [1.20, 2.03]. Interviewees found the unanticipated questions combined (M = 3.71, SD = 1.66) more cognitively demanding than the anticipated questions (M = 2.75, SD = 1.61), *F* = 16.98, *p* < .001, *d =* 0.58, 95% CI [0.19, 0.97]. Additionally, spatial/temporal questions (M = 4.15, SD = 1.70) were rated as significantly more cognitively demanding than planning questions (M = 3.28, SD = 1.52), *F* = 10.53, *p* = .002, *d* = 0.53, 95% CI [0.09, 0.98].

### Perceived cues

The reasons that the interviewers reported for their veracity decisions were grouped into four categories: verbal content, verbal delivery, non-verbal passive, and non-verbal active. The total number within each category was calculated for each interview. A multiple regression was performed using these frequencies as predictors with accuracy (judged via the 7-point veracity scale) as the dependent variable. Verbal content was entered alone in the first step of the model, given that the unanticipated questions approach relies on detecting differences in the verbal content of interviewees’ responses [8], and verbal delivery, non-verbal passive and non-verbal active were entered together at the second step (see Table 2). The model at Step 1 was significantly able to predict interviewer accuracy, *F* (1, 118) = 6.22, *p* = .01, $\eta_{p}^{2}=.05$, 95% CI [.002, .14]. The greater the number of verbal content reasons that interviewers claimed to base their decisions on, the greater their accuracy level was. This provides support for Hypothesis 3. Despite this, the model was only able to explain 5% of the variance in accuracy. The model remained significant at Step 2, *F* (4, 115) = 2.89, *p* = .03, $\eta_{p}^{2}=.09$, 95% CI [.001, .17], however, the addition of the three remaining predictors did not significantly improve the model, Δ*R*^2^ = .04, *F* (3, 115) = 1.74, *p* = .16. Inspection of the data for Step 2 reveals that verbal delivery, and both non-verbal categories were negatively related to interviewer accuracy, indicating that the more of these types of reasons that interviewers based their decisions on, the worse their accuracy became. However, none of these were significant predictors.

**Table 2:** Regression outcome for post-interview veracity decision (truth-teller versus liar) made by interviewers.

	*b*	SE *b*	*β*	95% CI for *b*	
				Lower	Upper
**Step 1**					
Constant	3.49	0.31		2.89	4.09
Verbal Content	0.50	0.20	.22*	0.10	0.90
**Step 2**					
Constant	4.25	0.45		3.36	5.14
Verbal Content	0.38	0.21	.17	-0.02	0.79
Verbal Delivery	-0.38	0.28	-.13	-0.92	0.17
Non-verbal Passive	-0.24	0.27	-.08	-0.77	0.30
Non-verbal Active	-0.23	0.14	-.15	-0.51	0.05

Note: *R*^2^ = .05 for Step 1 (*p* = .01), Δ*R*^2^ = .04 for Step 2 (*p* = .16).

* *p* < .05.

The analysis was repeated, with a binary logistic regression, using forced choice accuracy as the dependent variable. The findings were essentially the same as those of the Likert scale data. The model at Step 1 was significantly able to predict interviewer accuracy, χ^2^(1) = 4.07, *p =* .05. The greater the number of verbal content reasons that interviewers claimed to base their decisions on, the greater their accuracy levels. Despite this, the model was only able to explain 5% of the variance in accuracy (Nagelkerke *R*^2^). The model was no longer significant at step two. The addition of the three remaining predictors did not significantly improve the model, χ^2^(3) = 3.92, p = .27.

### Reality monitoring analysis

#### Analysis approach

The text analysis software programme Linguistic Inquiry and Word Count (LIWC, [43]) was used to carry out word counts in this study. In order to investigate the effects of veracity and question type, only transcripts of the final five questions in each interview were included in analysis (the first five being common to all conditions). To prepare the transcripts for analysis, all utterances from the interviewer were removed, leaving only responses made by interviewees. Responses from each interview (including utterances, such as ‘er’ or ‘hmm’) were entered together as one paragraph. Filler words, such as ‘you know’, were transcribed as one word (e.g., ‘youknow’). Finally, the word ‘like’, when used as a filler word, was transcribed as ‘rrlike’ in order to be classified as such by LIWC.

For each transcript, LIWC determines the amount of words falling into 73 linguistic categories, each presented as percentages of total word count. Four of relevance to RM were analysed: ‘perceptual processes’, ‘relativity’, ‘affective processes’, and ‘cognitive mechanisms’. The ‘perceptual processes’ (or ‘sensory’) category includes words relating to sensory information, such as ‘saw’, ‘heard’, and ‘felt’. The ‘relativity’ (or ‘contextual’) category includes spatial and temporal related words, such as ‘down’, ‘arrive’, and ‘during’. The ‘affective processes’ category includes emotion-based words, both positive and negative, such as ‘happy’, ‘hurt’, and ‘worried’. Finally, the ‘cognitive mechanisms’ category includes words associated with thought processes, such as ‘know’, ‘think’, ‘maybe’ and ‘because’. These categories are similar to those used by Bond et al [44].

#### Word count

**Fig 3:**
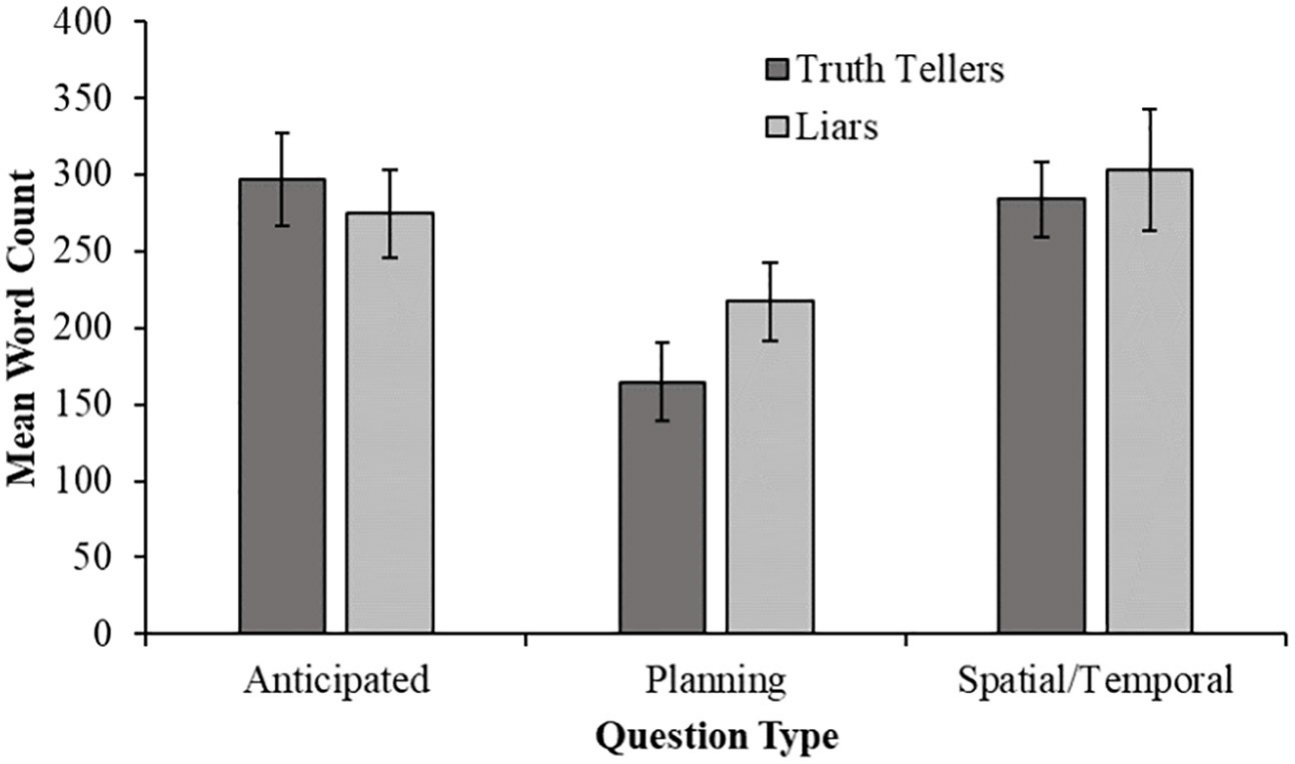
Mean word count of the responses provided by interviewees. Error bars represent +/-1 SEM.

In order to explore the effects of veracity and question type on the total number of words spoken by interviewees, a 2 (Veracity: truth-teller or liar) × 3 (Question Type: anticipated, unanticipated planning, or unanticipated spatial/temporal) ANOVA was conducted with word count as the dependent variable (Fig 3). There was no effect of veracity, *F* (1, 114) = 0.45, *p* = .50, nor was there a significant interaction, *F* (2, 114) = 0.81, *p* = .45. However, there was a significant main effect of question type, *F* (2, 114) = 7.52, *p* = .001, $\eta_{p}^{2}=.12$, 95% CI [.02, .22]. Post-hoc tests revealed a significantly lower word count in response to unanticipated planning questions (M = 190.88, SD = 115.54) than to both anticipated questions (M = 285.98, SD = 130.62), *t* (78) = -3.45, *p* = .001, *d* = 0.77, 95% CI [0.31, 1.22] and unanticipated spatial/temporal questions (M = 293.23, SD = 145.81), *t* (78) = -3.48, *p* = .001, *d* = 0.78, 95% CI [0.32, 1.23]. There was no significant difference in word count between responses to anticipated questions and unanticipated spatial/temporal questions, *t* (78) = -0.23, *p* = .82.

#### Category counts

Table 3 shows the mean percentage of truth-tellers’ and liars’ statements in each RM category for each of the three question types. To examine the effects of veracity and question type, a 2 (Veracity: truth-teller or liar) × 3 (Question Type: anticipated, unanticipated planning, or unanticipated spatial/temporal) MANOVA was conducted with the four RM criteria entered as dependent variables. There were significant overall main effects of veracity, *F* (4, 111) = 2.59, *p* = .04, $\eta_{p}^{2}=.09$, 95% CI [.001, .17], and question type, *F* (8, 224) = 16.10, *p* < .001, $\eta_{p}^{2}=.37$, 95% CI [.25, .43]. Additionally, there was a significant veracity × question type interaction, *F* (8, 224) = 2.01, *p* = .046, $\eta_{p}^{2}=.07$, 95% CI [.001, .11].

**Table 3:** RM category mean (SD) counts for each question type.

	Truth tellers	Liars
**Anticipated Questions**		
Sensory	2.30 (1.38)	1.51 (0.96)
Contextual	20.77 (3.60)	19.68 (4.03)
Affective	2.09 (0.86)	2.25 (1.25)
Cognitive Mechanisms	8.82 (2.22)	8.94 (2.01)
**Planning Questions**		
Sensory	1.55 (1.83)	1.93 (1.15)
Contextual	17.20 (5.41)	18.01 (3.64)
Affective	1.89 (1.27)	3.07 (1.27)
Cognitive Mechanisms	16.28 (4.38)	17.80 (3.30)
**Spatial and Temporal Questions**		
Sensory	1.90 (1.00)	1.32 (0.57)
Contextual	22.64 (5.26)	23.99 (5.60)
Affective	1.59 (0.87)	1.92 (1.05)
Cognitive Mechanisms	8.53 (4.02)	7.96 (2.90)

Univariate tests of the four RM criteria revealed a significant effect of veracity with affective words, *F* (1, 114) = 7.59, *p* = 0.01, $\eta_{p}^{2}=.06$, 95% CI [.01, .16], showing that liars (M = 2.41, SD = 1.27) used significantly more affective words than truth tellers (M = 1.86, SD = 1.01), *t* (118) = -2.65, *p* = .01, *d* = 0.48, 95% CI [0.12, 0.85]. The effects of veracity with the remaining RM criteria were not significant (all *p*s > .05).

There was a significant univariate effect of question type on contextual words, *F* (2, 114) = 15.00, *p* < .001, $\eta_{p}^{2}=.21$, 95% CI [.08, .32], with significantly more in response to unanticipated spatial/temporal questions (M = 23.32, SD = 5.40) than to both anticipated questions (M = 20.22, SD = 3.81), *t* (78) = 2.96, *p* = .004, *d* = 0.66, 95% CI [0.21, 1.11], and unanticipated planning questions (M = 17.61, SD = 4.57), *t* (78) = 5.10, *p* < .001, *d* = 1.14, 95% CI [0.66, 1.61]. There was also an effect of question type on affective words, *F* (2, 114) = 4.33, *p* = .02, $\eta_{p}^{2}=.07$, 95% CI [.003, .16], with significantly more in response to unanticipated planning questions (M = 2.48, SD = 1.39) than to unanticipated spatial/temporal questions (M = 1.75, SD = 0.97), *t* (78) = 2.72, *p* = .01, *d* = 0.61, 95% CI [0.16, 1.05]. Finally, there was a significant effect of question type on cognitive mechanism words, *F* (2, 114) = 90.67, *p* < .001, $\eta_{p}^{2}=.61$, 95% CI [.50, .69], with significantly more in response to unanticipated planning questions (M = 17.04, SD = 3.90) than to both anticipated questions (M = 8.88, SD = 2.10), *t* (78) = 11.65, *p* < .001, *d* = 2.61, 95% CI [2.00, 3.20], and to unanticipated spatial/temporal questions (M = 8.24, SD = 3.47), *t* (78) = 10.64, *p* < .001, *d* = 2.38, 95% CI [1.80, 2.95]. There was no significant effect of question type on perceptual details, *F* (2, 114) = 0.60, *p* = .55.

## Discussion

The results of Experiment 1 indicate that the manipulations were successful. The planning and spatial/temporal questions were rated as significantly less anticipated than the anticipated questions. Additionally, participants appeared to comply with the instructions and were motivated to do so. As with all subjective response measures, responses to the post-interview questionnaire may have been influenced by study demand characteristic. Nonetheless, the absence of differences between conditions gives us some degree of confidence that the motivation to conform was high and equivalent across conditions. Overall, the findings of Experiment 1 indicate that unanticipated questions did not increase interviewers’ ability to detect interviewee veracity. The veracity scale judgements and forced choice results show the same pattern: while accuracy for detecting liars increased slightly with unanticipated questions, accuracy at detecting truth-tellers was reduced, particularly with planning questions. As such, the findings fail to support Hypothesis 1. Furthermore, the results do not support the idea that unanticipated questions raise cognitive load for liars but not for truth tellers, failing to support Hypothesis 2. The unanticipated questions approach is grounded in the idea that being asked unanticipated questions in an interview will raise the cognitive load for liars but not truth tellers [8]. However, in the present study, liars found the interviews more difficult than truth tellers regardless of question type, and all interviewees found the unanticipated spatial/temporal interviews more cognitively demanding than the anticipated or unanticipated planning interviews, regardless of veracity condition. There was, however, a small positive correlation between accuracy and the number of verbal content reasons interviewers claimed to base their veracity judgements on, supporting Hypothesis 3.

Previous research has shown that truth tellers use more words associated with sensory, contextual and affective processes than liars, while liars tend to use more cognitive mechanism words than truth tellers [40–42]. The present study found a difference in the number of affective words given by liars and truth tellers, providing modest support for Hypothesis 4. Truth-tellers and liars used qualitatively different language in response to the three question types, with more contextual detail words when answering the spatial/temporal questions and more cognitive mechanism words with planning questions. However, although a significant interaction was found between veracity and question type, at a univariate level there was no significant effect for any of the four RM categories, thus Hypothesis 5 was rejected. It seems that the content of unanticipated questions alters the content of answers, but not in a way that discriminates truth-tellers from liars.

## Experiment 2

For tasks such as security screeing and police stop-and-search interviews, methods are needed that can be used to determine interviewee veracity in real time. However, in other contexts, the ability to detect deception in a post-hoc fashion is also important. For example, UK police officers are trained according to the PEACE model of investigative interviewing, which states that the purpose of such interviews is to gather information for use by others rather than to determine guilt or innocence directly [45]. The information gathered by interviewers, including interview transcripts, may then be used by independent observers, such as judges and juries, in subsequent legal proceedings. Therefore, in Experiment 2, transcripts of the interviews gathered in Experiment 1 were shown to a group of observers who were required to make veracity judgements.

Experiment 1 found that interviewees used qualitatively different language in response to the three question types, with planning questions yeilding more references to cognitive operations and spatial/temporal questions yeilding more contextual words. Experiment 1 failed to support the unanticipated question approach in terms of its ability to allow interviewers to accurately determine the interviewees’ veracity. However, there was a positive relationship between interviewers’ reported reliance on verbal content when making veracity judgements and their accuracy. Despite this, the literature on detecting deception suggests that individuals rarely base decisions purely on verbal cues, and instead tend to focus on non-verbal behaviour such as eye contact, body movements, and general demeanour [2,3]. The interviewers in Experiment 1 often reported using such non-verbal indicators when making veracity judgements. As such, it is possible that poor accuracy rates could be attributed to interviewers relying on ineffective non-verbal cues [4], as opposed to more useful verbal cues elicited by unanticipated questions. Experiment 2 was conducted in order to determine whether unanticipated questions could improve veracity judgement accuracy when non-verbal behaviour is not present to influence decision making. Previous observer studies have reported positive results. For example, Vrij et al [30,31] found that observers were able to accurately determine the veracity of interviewees when the transcripts contained unanticipated questions, but not from transcripts containing only anticipated questions.

Based on these findings [30,31], as well as research into the unanticipated questions approach showing differences between truth-tellers’ and liars’ verbal behaviour [9,13,14], we expected to find that observers would show higher levels of accuracy when judging the veracity of transcripts containing unanticipated questions, compared to those containing anticipated questions (Hypothesis 6).

## Method

### Participants

Ninety females (M_*age*_ = 30.30, SD = 16.40) and 21 males (M_*age*_ = 34.62, SD = 17.78) took part in the study. Participants voluntarily took part in the experiment as part of an Open Day at the University of Sussex. All gave their informed consent to take part and were free to withdraw at any point. This study was approved by the Sciences & Technology Cross-Schools Research Ethics Committee at the University of Sussex.

### Design

A repeated measures design was employed. There were three different interview question types (anticipated, unanticipated planning, and unanticipated spatial/temporal), each answered by either a truth-teller or a liar, creating a total of six conditions. Each participant was presented with one randomly selected transcript from each of the six conditions.

### Procedure

Transcripts were taken from the interviews which took place during Experiment 1. Experiment 2 used transcripts of the final five questions in each interview. In order to moderate effects of variation in interviewee response length, the number of words used by the interviewee in each interview was analysed and the lowest and highest five in each of the six conditions were excluded, leaving ten transcripts per condition (see Table 4 for means).

**Table 4:** Mean (SD) word count of transcripts in each condition.

Question Type	Truth teller	Liar
Anticipated	268.10 (48.15)	260.80 (46.14)
Planning	154.90 (52.54)	222.40 (72.09)
Spatial and Temporal	263.10 (51.65)	262.70 (96.82)

Participants were informed that they would be reading interview transcripts in which the interviewee may have been telling the truth or lying. They were then told “after reading each transcript, you will be required to state whether you believe the person being interviewed was telling the truth or whether they were lying.” Before beginning, the participants were asked to read the instructions for the navigation task that participants received in Experiment 1. Participants were randomly presented on a computer screen with one of ten transcripts from each condition (i.e. they received six transcripts in total) and were given a maximum of three minutes to read each transcript. The order in which the six conditions appeared on screen was counter-balanced across participants. Following each transcript, they were asked to indicate whether they thought the interviewee was telling the truth or lying. This was done via both a seven point scale and a dichotomous forced choice decision.

## Results

### Accuracy

#### Forced choice

Observers made a dichotomous forced choice decision regarding the veracity of the interviewees in each of the transcripts. A series of one-sample t-tests were carried out to investigate effects of veracity and question type on observer accuracy (see Table 5). Looking at detection rates of liars and truth-tellers separately, accuracy at judging anticipated question transcripts was significantly better than chance when identifying truth-tellers, *t* (110) = 2.22, *p* = .03, *d* = 0.21, 95% CI [0.02, 0.40], but not liars, *t* (110) = -1.43, *p* = .16. When looking at truth-tellers and liars combined, the observer accuracy rate was not significantly greater than chance, *t* (110) = 0.55, *p* = .58.With unanticipated planning transcripts, performance did not significantly differ from chance when identifying truth-tellers, *t* (110) = 1.63, *p* = .11, or liars, *t* (110) = 1.63, *p* = .11, however, with truth-tellers and liars combined, the accuracy did exceed chance level, *t* (110) = 2.49, *p* = .014, *d* = 0.24, 95% CI [0.05, 0.42]. With unanticipated spatial/temporal transcripts, accuracy levels exceeded chance for both truth-tellers, *t* (110) = 3.71, *p* < .001, *d* = 0.35, 95% CI [0.16, 0.54], and liars, *t* (110) = 4.65, *p* < .001, *d* = 0.44, 95% CI [0.25, 0.64]. When looking at the accuracy rate of truth-tellers and liars combined, observer accuracy was again greater than chance level, *t* (110) = 5.78, *p* < .001, *d* = 0.55, 95% CI [0.35, 0.75].

**Table 5:** Mean (SD) observer accuracy rates across each question type for truth-tellers and liars.

Question Type	Truth-teller	Liar	Overall
Anticipated	**60%** (49%)	43% (50%)	52% (34%)
Planning	58% (50%)	58% (50%)	**58%** (32%)
Spatial/Temporal	**67%** (47%)	**70%** (46%)	**68%** (34%)

Note: Bold figures indicate that the accuracy significantly differed from chance (50%)

#### Veracity scale

**Fig 4:**
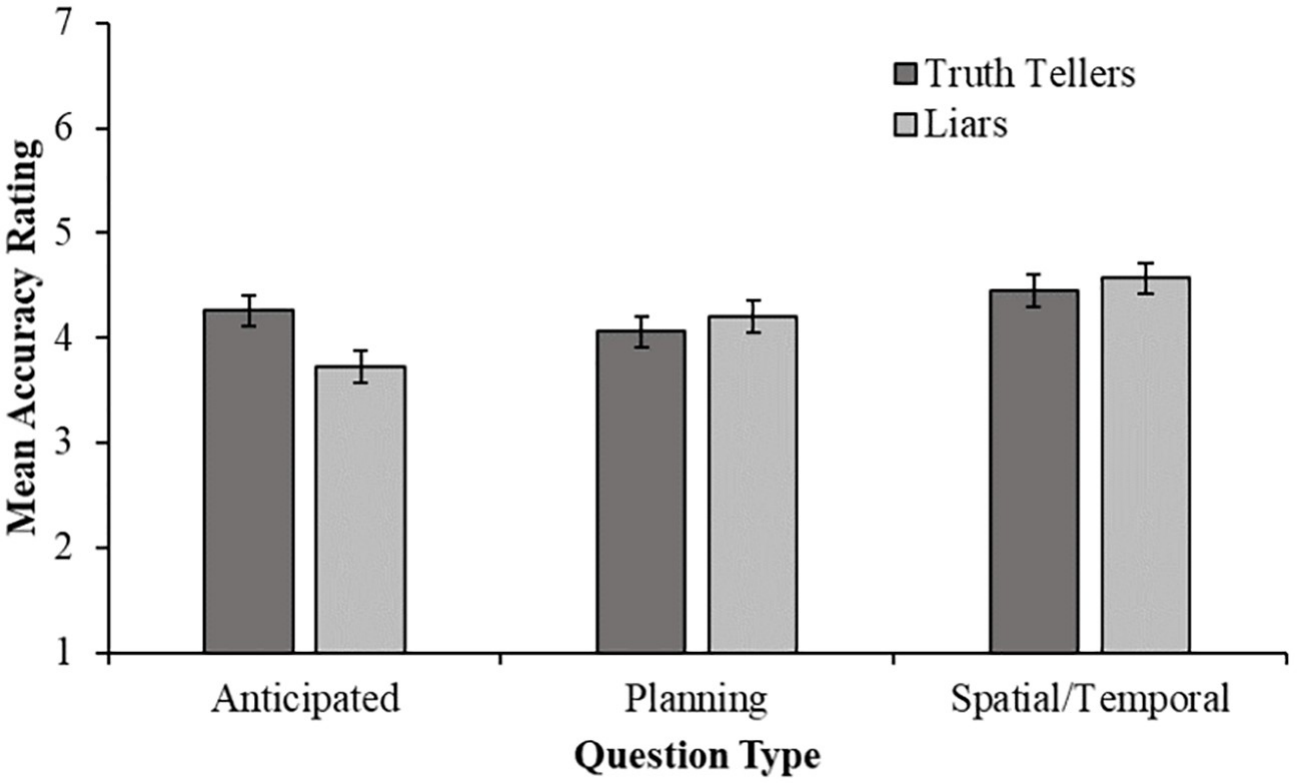
Mean observer accuracy (measured via 7-point Likert scale) across question type for truth-tellers and liars separately. Error bars represent +/- 1 SEM.

As well as making a dichotomous forced choice decision, observers were required to rate whether they thought the interviewee was telling the truth or lying on a seven point scale (1 = definitely lying; 7 = definitely telling the truth). Scores given to transcripts in the lying condition were reversed meaning that higher scores indicate greater accuracy across all trials. Fig 4 shows the mean scores given across the three question types for truth-tellers and liars.

A two way 2 (veracity: truth-teller or liar) × 3 (question type: anticipated, unanticipated planning, or unanticipated spatial/temporal) repeated measures ANOVA was performed on rating accuracy. There was no significant main effect of veracity, *F* (1, 110) = 0.64, *p* = .43, nor was there a significant interaction, *F* (2, 220) = 2.88, *p* = .06. However, there was a significant main effect of question type, *F* (2, 220) = 6.32, *p* = .002, $\eta_{p}^{2}=.05$, 95% CI [.008, .12]. Planned contrasts revealed that accuracy was significantly greater when observers were judging the transcripts of unanticipated questions (i.e. planning and spatial/temporal questions combined) compared to anticipated questions, *F* (1, 110) = 6.53, *p* = .01, $\eta_{p}^{2}=.06$, 95% CI [.003, .16]. Furthermore, observer accuracy was significantly higher when judging the spatial/temporal questions than the planning questions, *F* (1, 110) = 6.13, *p =* .02, $\eta_{p}^{2}=.05$, 95% CI [.002, .15].

## Discussion

The findings of Experiment 2 provide only partial support for the unanticipated questions approach [7], and suggest that the type of question asked is crucial. In forced choice judgements, accuracy was greatest when observers were reading transcripts of interviews that included unanticipated spatial/temporal questions. Accuracy when judging transcripts of the anticipated questions was marginally better than chance for truth-tellers, but not liars. When judging the transcripts of planning questions, accuracy was not above chance for truth tellers or liars. When using a scale to make veracity judgements, observer accuracy was greater when judging unanticipated questions than anticipated questions. This is in line with the findings of Vrij et al [30,31], providing some support for Hypothesis 6, as well as the unanticipated questions approach generally. However, accuracy was also shown to be higher when observers were judging transcripts of unanticipated spatial/temporal questions compared to transcripts of unanticipated planning questions, which indicates that the type of unanticipated questions asked can have an impact on the ability to determine interviewee veracity.

## General discussion

Two experiments explored the effects of different types of unanticipated question on interviewer and observer veracity judgements, and on the content of interviewee accounts. Experiment 1 compared anticipated, unanticipated planning and unanticipated spatial/temporal questions in investigative interviews, with a focus on interviewers’ veracity judgement accuracy. The findings fail to provide support for the unanticipated questions approach. With dichotomous forced-choice judgements, accuracy for truth-tellers was no greater when interviewers were asking unanticipated planning or spatial/temporal questions than when asking the anticipated questions. For liars, interviewers were more accurate when asking unanticipated compared to anticipated questions, though neither question type yielded accuracy rates significantly greater than chance. With veracity scale judgements, there was no effect of question type. Accuracy was greater for truth-tellers than liars overall, but this difference was diminished when interviewers asked unanticipated questions compared to the anticipated questions. This suggests that the unanticipated questions approach was marginally useful in improving the detection of liars, but impaired the detection of truth-tellers.

According to Vrij [8], unanticipated questions raise the cognitive load for liars but not for truth-tellers, resulting in observable verbal cues to deceit. In the present study, liars found the interviews more cognitively demanding than truth-tellers. However, all participants found answering unanticipated questions to be more cognitively demanding than anticipated questions, regardless of veracity condition. This suggests that, while lying is inherently more difficult than telling the truth, the use of unanticipated questions increased the cognitive load faced by liars and truth tellers equally. Previous studies have found similar results, with no interaction between veracity and question type [9,28]. This finding brings into question the proposed underlying mechanism of the unanticipated questions approach. Whatever differences there are between truth tellers and liars in their verbal behaviours when answering unanticipated questions, these differences may not be attributable to an increase in cognitive load faced exclusively by liars.

Experiment 1 also revealed that verbal content reasons for veracity decisions were positively associated with judgement accuracy. Verbal content can be a reliable indicator of veracity [4] and the unanticipated questions approach elicits verbal cues [7]. Despite this, the relationship between verbal content and accuracy was small, and the model could only account for 5% of the accuracy variance. Other variables appear to have contributed to accuracy, such as truth bias exhibited in the veracity judgements made by the interviewers. As with all studies of investigative interviews, the extent to which hypothesised base rates of expected truth-tellers and deceivers affected results cannot be assessed. In the present study, interviewers were given no information concerning the base rates fro truth-tellers and liars, and this might explain the appearance of a truth bias in interviewer responses. However, the absensce of differences between conditions in the presence of truth bias suggests that any impact of underlying base rates was independent of the effects of unanticipated questions. Though, as a result of this bias, accuracy was greater when detecting truth-tellers than liars across all question types, although not at ceiling. The interviewers in Experiment 1 received training. However, none were professionals within the criminal justice system. Novice veracity judges tend to be biased towards believing an interviewee’s account [46,47]. It is difficult to control for truth bias. One potential method for future studies would be to inform interviewers in advance that such bias is common. Research into prejudice shows that, by informing an individual of their implicit biases, they are capable of compensating for them [48]. Given that one of the aims of this paper was to assess the efficacy of unanticipated questions in terms of real-time veracity judgements, it should be noted that in genuine investigative scenarios these judgements would usually be conducted by trained professionals and, therefore, future studies may wish to investigate the potential effects that training and expertise might have on performance.

The RM analysis of Experiment 1 found an effect of veracity on affective words, with liars using more than truth-tellers. However, differences in the verbal content of truth tellers’ and liars’ transcripts were not increased by unanticipated questions. These findings do not support claims that unanticipated questions elicit differences in the verbal behaviour of truth-tellers and liars [7]. However, effects of question type were found, with contextual words arising more when answering unanticipated spatial/temporal questions and cognitive mechanism words arising more in responses to unanticipated planning questions. These findings indicate that the type of unanticipated question asked can have a significant effect on the type of information gathered. This may have important implications for determining interviewee veracity. If asking questions about planning taps into an individual’s cognitive operations, this may sometimes benefit liars. According to Oberlader et al [39], liars do not have a genuine perceptual experience of an event to base their responses on and must instead rely on their endogenously conceived, imagined experiences of the event. By asking questions that require introspective consideration and result in responses rich in information related to cognitive mechanisms concerning judgement (e.g., estimations) or decision making (e.g., hypothesising), the interviewer may be providing a liar with a framework with which to provide a plausible answer.

On a positive note, previous advantages of unanticipated questioning for observer judgements were confirmed in Experiment 2, particularly with unanticipated questions that focussed on spatial/temporal details. Moreover, the findings of the dichotomous decisions showed that, in line with the results of Experiment 1, the advantages of asking unanticipated questions was more evident for the detection of liars. The increase in cognitive load experienced by truth-tellers raises the concern that, if used in practical settings, insensitive use of unanticipated questioning may increase the likelihood of mistaking truth-tellers for liars. Spatial/temporal questions emphasise differences in the ways in which truth-tellers and liars use contextual words; planning questions that encourage the discussion of cognitive operations do not.

Taken together, the results of the studies provide little support for the unanticipated questions approach to veracity testing. There is some support for the approach in a post-interview observer scenario, though it appears that some forms of unanticipated question will be more successful in this situation than others. Furthermore, the cognitive load explanation provided by Vrij [8] was refuted, leading to potential concerns regarding the application of the approach in practical settings.

## Supporting information

S1 Appendix Question used for anticipated, unanticipated planning and unicipated spatial and temporal conditions. (DOCX)

S2 Appendix Post-interview questionnaire given to the interviewer. (DOC)

S3 Appendix Post-interview questionnaire given to the interviewee. (DOCX)

S4 Appendix Post-interview question anticipation questionnaire given to interviewees in the anticipated question condition. (DOCX)

S5 Appendix Post-interview question anticipation questionnaire given to interviewees in the planning question condition. (DOCX)

S6 Appendix Post-interview question anticipation questionnaire given to interviewees in the spatial/temporal question condition. (DOCX)
