# Supplementary material for: Unanticipated questions can yield unanticipated outcomes in investigative interviews
Source: PLoS One. 2018 Dec 7;13(12):e0208751. doi: 10.1371/journal.pone.0208751 (PMC6285978; doi:10.1371/journal.pone.0208751)
Supplement: S1 Appendix — (DOCX) [file pone.0208751.s001.docx]

**Question list A (Anticipated)**

1. “What task did you carry out around the campus today?”
2. “How many boxes were in room A when you arrived there?”
3. “Describe the route you took from building A to building B.”
4. “Who let you in to building B?”
5. “Describe the items that you collected from building B.”
6. “How many boxes were there in the room at building B?”
7. “How difficult was the task to carry out?”
8. “Describe any discussion you had with the experimenter whilst at building B.”
9. “In relation to building B, how familiar are you with that area of the campus?”
10. “Please describe the task one final time, from start to finish. Try to be as detailed as possible.”

**Question list B (Planning)**

1. “What task did you carry out around the campus today?”
2. “How many boxes were in room A when you arrived there?”
3. “Describe the route you took from building A to building B.”
4. “Who let you in to building B?”
5. “Describe the items that you collected from building B.”
6. “What was the main goal of your planning?”
7. “What was the final thing you planned?”
8. “What was the most difficult part of your planning?”
9. “Explain what steps you would have taken had you not been able to access building B via the main door.”
10. “Please describe any changes you made to your plan during the planning stage.”

**Question list C (Spatial/Temporal)**

1. “What task did you carry out around the campus today?”
2. “How many boxes were in room A when you arrived there?”
3. “Describe the route you took from building A to building B.”
4. “Who let you in to building B?”
5. “Describe the items that you collected from building B.”
6. “In relation to building B, try to imagine the layout and features of the room where you collected the boxes from. Please describe this room to me, and be as detailed as you can.”
7. “In building B, where were the boxes in relation to the door you entered through?”
8. “How long did it take to walk from building A to building B?”
9. “In relation to building B, other than the experimenter, where was the closest other person as you left the building?”
10. “Please describe the task in full one last time, but now in reverse order. Try to be as detailed as possible.”
