# Supplementary material for: Unanticipated questions can yield unanticipated outcomes in investigative interviews
Source: PLoS One. 2018 Dec 7;13(12):e0208751. doi: 10.1371/journal.pone.0208751 (PMC6285978; doi:10.1371/journal.pone.0208751)
Supplement: S2 Appendix — (DOC) [file pone.0208751.s002.doc]

Interviewer Questionnaire

Interviewer Number:

Question List:

1. Do you think that the interviewee was telling the truth or lying?

2. How confident are you that your judgment about whether or not the interviewee was telling the truth or lying is correct?

3. At what point did you decide whether the interviewee was telling the truth or lying?

4. How difficult did you find it to decide whether the interviewee was telling the truth or lying?

5. Please explain **why** you found it easy/difficult to decide whether the interviewee was telling the truth/lying.

___________________________________________________________________________________________________________________________________________________________________________________________________________________________________________________________________________________________________________________________________________________________________________________________________________________________________________________________________________________________________

6. What type of information did you use to decide whether the interviewee was telling the truth or lying?

7. Please explain/describe the type of information you used to decide whether the interviewee was telling the truth or lying. For example, if you indicated in Q6 that you used both verbal and non-verbal behaviour, please describe/explain those behaviours in as much detail as possible.

________________________________________________________________________________________________________________________________________________________________________________________________________________________________________________________________________________________________________________________________________________________________________________________________________________________________________________________________________________________________________________________________________________________________________

_____________________________________________________________________

8. If you had to make a firm decision, would you say the interviewee was telling the truth or lying? Please tick the appropriate box.

**Lying Telling Truth**

9. Please provide a further question you could have asked, that you believe is both relevant to the interview and which would **not** have been anticipated by the interviewee (please try to provide a new question each time you complete this section).

________________________________________________________________________________________________________________________________________________________________________________________________________________________________________________________________________________________________________________________________________________________

____________________________________________________________________

____________________________________________________________________

10. Please write below any comments/suggestions you may have concerning your participation in this research.

________________________________________________________________________________________________________________________________________________________________________________________________________________________________________________________________________________________________________________________________________________________

**Thank you for participating in this study.**
