# Supplementary material for: Unanticipated questions can yield unanticipated outcomes in investigative interviews
Source: PLoS One. 2018 Dec 7;13(12):e0208751. doi: 10.1371/journal.pone.0208751 (PMC6285978; doi:10.1371/journal.pone.0208751)
Supplement: S3 Appendix — (DOCX) [file pone.0208751.s003.docx]

1

| **Participant No:** | **Post Interview Questionnaire** | **Condition:** |
| --- | --- | --- |
|  |  |  |
| 1. Age ________ | 2. Gender __________________ |  |

1. Occupation (if student please state whether UG or PG) ___________________________
2. On a scale from 1 to 7 please rate **how deceptive/truthful** you were during the interview.

|  |  |  |  |  |  | **Totally** |
| --- | --- | --- | --- | --- | --- | --- |
| **Totally** |  |  | **Somewhat** |  |  | **deceptive** |
| **truthful** |  |  | **deceptive** |  |  | (everything I |
| (everything I |  |  | (about half of |  |  | told the |
| told the |  |  | what I told the |  |  | interviewer |
| interviewer |  |  | interviewer |  |  | was |
| was truthful) |  |  | was deceptive) |  |  | deceptive) |
| 1 | 2 | 3 | 4 | 5 | 6 | 7 |
| □ | □ | □ | □ | □ | □ | □ |

1. On a scale from 1 to 7 please rate **how difficult/cognitively demanding** you found the interview.

| **Very easy/** |  |  | **Somewhat** |  |  | **Very** |
| --- | --- | --- | --- | --- | --- | --- |
| **not at all** |  |  | **difficult/** |  |  | **difficult/** |
| **cognitively** |  |  | **cognitively** |  |  | **cognitively** |
| **demanding** |  |  | **demanding** |  |  | **demanding** |
| 1 | 2 | 3 | 4 | 5 | 6 | 7 |
| □ | □ | □ | □ | □ | □ | □ |

1. **Prior** to the interview, in order to convince the interviewer that you were telling the truth about your account, to what extent did you think about what you would **say** in the interview?

| **I did not** |  |  |  |  |  |  |
| --- | --- | --- | --- | --- | --- | --- |
| **think about** |  |  | **I gave some** |  |  | **I thought a** |
| **what I** |  |  | **thought to** |  |  | **lot about** |
| **would say** |  |  | **what I would** |  |  | **what I** |
| **at all** |  |  | **say** |  |  | **would say** |
| 1 | 2 | 3 | 4 | 5 | 6 | 7 |
| □ | □ | □ | □ | □ | □ | □ |

2

1. If you **did** have a strategy concerning what you would **say** please describe it. Be as detailed as possible and include an explanation as to why you decided upon this strategy (later questions will be asking you about your behaviour during the interview, so this question is only about what you planned to **say** during the interview).

______________________________________________________________________

______________________________________________________________________

______________________________________________________________________

______________________________________________________________________

______________________________________________________________________

______________________________________________________________________

______________________________________________________________________

______________________________________________________________________

______________________________________________________________________

______________________________________________________________________

1. If, before the interview, you did **not** devise a strategy concerning what you would **say** please explain why **not.**

______________________________________________________________________

______________________________________________________________________

______________________________________________________________________

______________________________________________________________________

______________________________________________________________________

1. If you **did** devise a strategy concerning what you would **say,** to what extent did you actually **use** this strategy during the interview?

| **I did not** |  |  |  |  |  | **I used my** |
| --- | --- | --- | --- | --- | --- | --- |
| **use my** |  |  | **I used my** |  |  | **strategy to** |
| **strategy at** |  |  | **strategy to** |  |  | **its full** |
| **all during** |  |  | **some extent** |  |  | **extent** |
| **the** |  |  | **during the** |  |  | **during the** |
| **interview** |  |  | **interview** |  |  | **interview** |
| 1 | 2 | 3 | 4 | 5 | 6 | 7 |
| □ | □ | □ | □ | □ | □ | □ |

3

1. **Prior** to the interview, in order to convince the investigator you were telling the truth, to what extent did you think about how you would **act and/or behave** during the interview?

| **I did not** |  |  |  |  |  |  |
| --- | --- | --- | --- | --- | --- | --- |
| **think about** |  |  | **I gave some** |  |  | **I thought a** |
| **how I would** |  |  | **thought to** |  |  | **lot about** |
| **behave at** |  |  | **how I would** |  |  | **how I would** |
| **all** |  |  | **behave** |  |  | **behave** |
| 1 | 2 | 3 | 4 | 5 | 6 | 7 |
| □ | □ | □ | □ | □ | □ | □ |

1. If you **did** have a strategy concerning how you planned to **behave** please describe it. Be as detailed as possible and include an explanation as to why you decided upon this strategy.

______________________________________________________________________

______________________________________________________________________

______________________________________________________________________

______________________________________________________________________

______________________________________________________________________

______________________________________________________________________

1. If, before the interview, you did **not** devise a strategy, concerning how you would **behave,** please explain why **not**.

______________________________________________________________________

______________________________________________________________________

______________________________________________________________________

______________________________________________________________________

______________________________________________________________________

4

1. If you **did** have a strategy concerning how you would **behave,** to what extent did you actually use this strategy during the interview?

| **I did not** |  |  |  |  |  | **I used my** |
| --- | --- | --- | --- | --- | --- | --- |
| **use my** |  |  | **I used my** |  |  | **strategy to** |
| **strategy at** |  |  | **strategy to** |  |  | **its full** |
| **all during** |  |  | **some extent** |  |  | **extent** |
| **the** |  |  | **during the** |  |  | **during the** |
| **interview** |  |  | **interview** |  |  | **interview** |
| 1 | 2 | 3 | 4 | 5 | 6 | 7 |
| □ | □ | □ | □ | □ | □ | □ |

1. During the interview, on a scale from 1 to 7, **how motivated** were you to comply with the pre interview instructions?

| **Not at all** |  |  | **Somewhat** |  |  | **Very** |
| --- | --- | --- | --- | --- | --- | --- |
| **motivated** |  |  | **motivated** |  |  | **motivated** |
| 1 | 2 | 3 | 4 | 5 | 6 | 7 |
| □ | □ | □ | □ | □ | □ | □ |

1. Please write, below, further comments concerning your participation in this research.

______________________________________________________________________

______________________________________________________________________

______________________________________________________________________

______________________________________________________________________

______________________________________________________________________

______________________________________________________________________

______________________________________________________________________

______________________________________________________________________

______________________________________________________________________

**Thank you for participating in this study.**
