# Supplementary material for: Unanticipated questions can yield unanticipated outcomes in investigative interviews
Source: PLoS One. 2018 Dec 7;13(12):e0208751. doi: 10.1371/journal.pone.0208751 (PMC6285978; doi:10.1371/journal.pone.0208751)

**Difficulty and Anticipation Questionnaire B**

To what extent did you expect to be asked Question 1: “What task did you carry out around the campus today?”


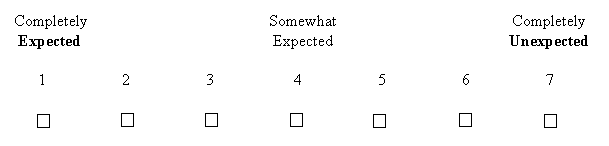


To what extent did you expect to be asked Question 2 “How many boxes were in room A when you arrived there?”


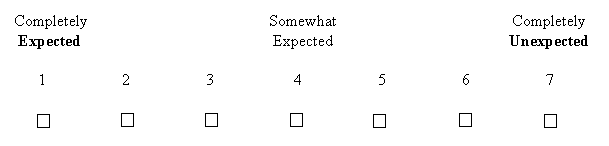


To what extent did you expect to be asked Question 3: “Describe the route you took from building A to building B”


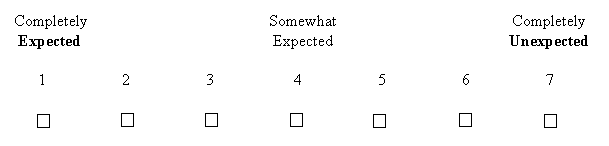


To what extent did you expect to be asked Question 4: “Who let you in to building B?”


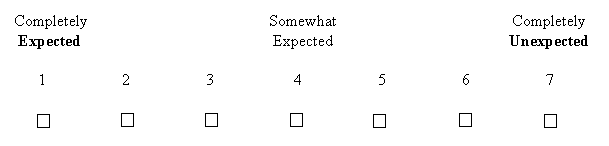


To what extent did you expect to be asked Question 5: “Describe the items that you collected from building B.”


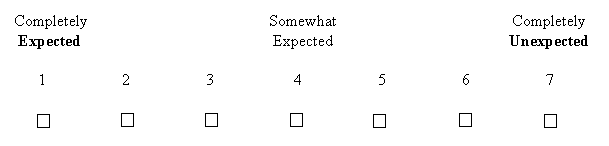


To what extent did you expect to be asked Question 6: “What was the main goal of your planning?”


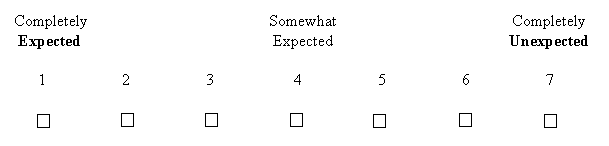


To what extent did you expect to be asked Question 7: “What was the final thing you planned?”


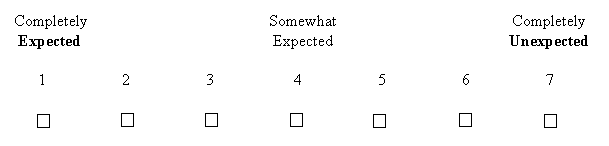


To what extent did you expect to be asked Question 8: “What was the most difficult part of your planning?”


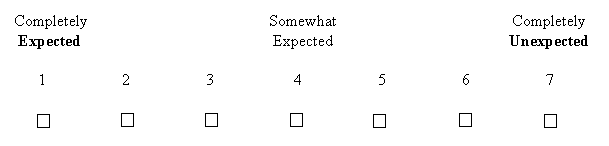


To what extent did you expect to be asked Question 9: “Explain what steps you would have taken had you not been able to access building B via the main door”


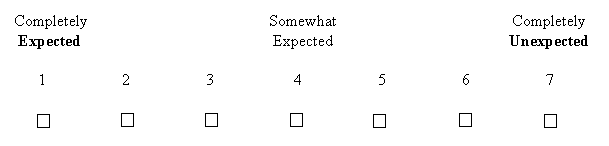


To what extent did you expect to be asked Question 10: “Please describe any changes you made to your plan during the planning stage”


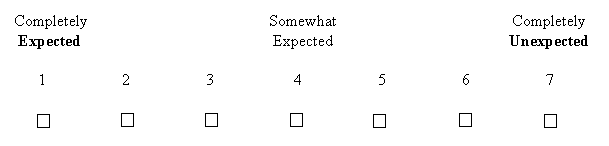

Supplement: S5 Appendix — (DOCX) [file pone.0208751.s005.docx]
