# Supplementary material for: Unanticipated questions can yield unanticipated outcomes in investigative interviews
Source: PLoS One. 2018 Dec 7;13(12):e0208751. doi: 10.1371/journal.pone.0208751 (PMC6285978; doi:10.1371/journal.pone.0208751)

**Difficulty and Anticipation Questionnaire C**

To what extent did you expect to be asked Question 1: “What task did you carry out around the campus today?”


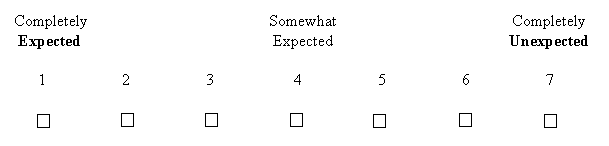


To what extent did you expect to be asked Question 2 “How many boxes were in room A when you arrived there?”


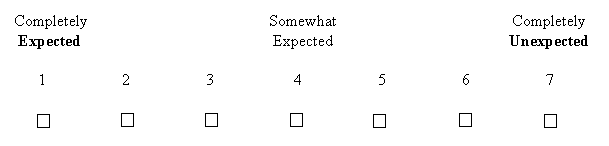


To what extent did you expect to be asked Question 3: “Describe the route you took from building A to building B”


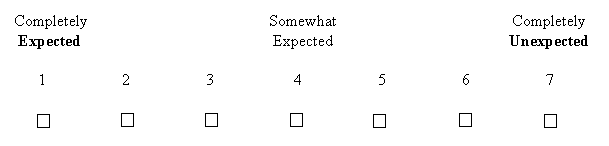


To what extent did you expect to be asked Question 4: “Who let you in to building B?”


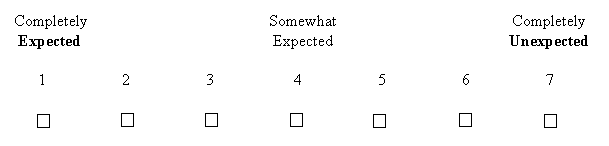


To what extent did you expect to be asked Question 5: “Describe the items that you collected from building B.”


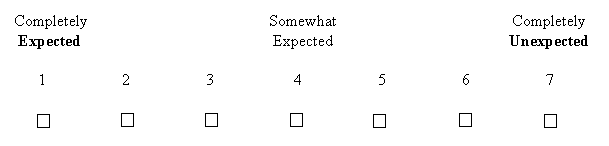


To what extent did you expect to be asked Question 6: “In relation to the first building you went to, try to imagine the layout and features of the room where you collected the package from. Please describe this room to me, and be as detailed as you can.”


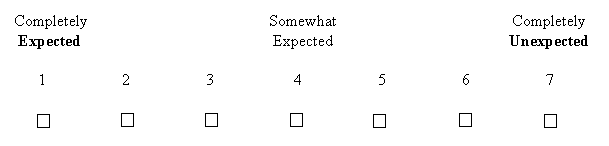


To what extent did you expect to be asked Question 7: “In building B, where were the boxes in relation to the door you entered through?”


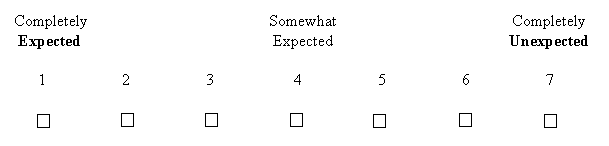


To what extent did you expect to be asked Question 8: “How long did it take to walk from building A to building B?”


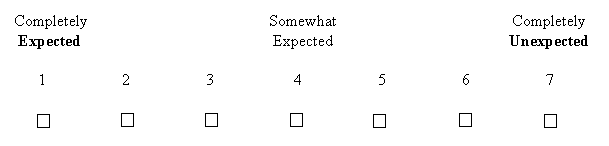


To what extent did you expect to be asked Question 9: “In relation to building B, other than the experimenter, where was the closest other person as you left the building?”


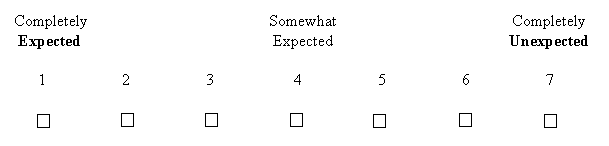


To what extent did you expect to be asked Question 10: “Please describe the task in full one last time, but now in reverse order. Try to be as detailed as possible”


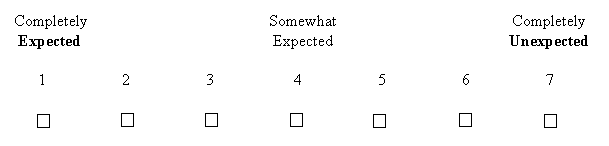

Supplement: S6 Appendix — (DOCX) [file pone.0208751.s006.docx]
